# Tool-use: An open window into body representation and its plasticity

**DOI:** 10.1080/02643294.2016.1167678

**Published:** 2016-06-17

**Authors:** Marie Martel, Lucilla Cardinali, Alice C. Roy, Alessandro Farnè

**Affiliations:** ^a^ Laboratoire Dynamique du Langage, CNRS UMR 5596, Lyon69007, France; ^b^ University of Lyon, Lyon69000, France; ^c^ The Brain and Mind Institute, University of Western Ontario, London, ON, Canada; ^d^ Integrative Multisensory Perception Action & Cognition team (ImpAct), Lyon Neuroscience Research Center, INSERM U1028, CNRS UMR5292, Lyon69000, France; ^e^ Hospices Civils de Lyon, Mouvement et Handicap & Neuro-immersion, Lyon69000, France

**Keywords:** Tool-use, body schema, body image, peripersonal space, kinematics

## Abstract

Over the last decades, scientists have questioned the origin of the exquisite human mastery of tools. Seminal studies in monkeys, healthy participants and brain-damaged patients have primarily focused on the plastic changes that tool-use induces on spatial representations. More recently, we focused on the modifications tool-use must exert on the sensorimotor system and highlighted plastic changes at the level of the body representation used by the brain to control our movements, i.e., the Body Schema. Evidence is emerging for tool-use to affect also more visually and conceptually based representations of the body, such as the Body Image. Here we offer a critical review of the way different tool-use paradigms have been, and should be, used to try disentangling the critical features that are responsible for tool incorporation into different body representations. We will conclude that tool-use may offer a very valuable means to investigate high-order body representations and their plasticity.

## Introduction

Performing accurate and efficient movements implies that the brain can access and integrate neural representations of both the body and the space around it. This integrated processing is even more challenging in the case of actions performed with tools, as one needs to transfer the control of the body to that of a mechanical effector. Humans have come to an exquisite level of mastery in tool-use, as dramatically witnessed when brain damage breaks this capability down, leading to apraxic deficits. While the cognitive motor and neural processes that allow for accurate tool-use have been extensively studied (Buxbaum, Shapiro, & Coslett, 2014; Goldenberg & Spatt, 2009; Hermsdörfer, Li, Randerath, Roby-Brami, & Goldenberg, 2013; Osiurak & Massen, 2014; Tarhan, Watson, & Buxbaum, 2015), in the present review we focus on the possibility that part of tool-mastery may emerge from the capability of incorporating tools into body representations (BR), as postulated by several theories (Arbib, Bonaiuto, Jacobs, & Frey, 2009; Cardinali et al., 2012; Farnè, Serino, & Làdavas, 2007; Jacobs, Bussel, Combeaud, & Roby-Brami, 2009; Maravita & Iriki, 2004). Many different approaches have been used to address the issue of how tools get incorporated into neural representations. Since the seminal monkey electrophysiology work by Iriki and colleagues (Iriki, Tanaka, & Iwamura, 1996), tool-use has been repeatedly reported to cause transient changes in a spatial representation termed peripersonal space (PPS). As it has recently become clear that tool-use may also affect bodily representations (Cardinali, Frassinetti, et al., 2009), a certain degree of confusion remains on how tool incorporation can and should be investigated as a function of the nature of the target brain representation. Here, for sake of completeness and because the historical focus has been put on remapping space with tools, we start by recalling some basic aspects of the peripersonal space leading to its plasticity. We then introduce some of the body representations that, across different dyadic and triadic models, are relevant to the purpose of this review and try to identify the conditions under which their plasticity becomes apparent following tool-use tasks.

## 1. Peripersonal space: A region of multisensory integration

Peripersonal space (PPS), defined as the space immediately surrounding the body, is now well accepted as a region of integration of somatosensory, visual, and auditory information (Graziano & Cooke, 2006; Rizzolatti, Fadiga, Fogassi, & Gallese, 1997). It is a privileged interface for interaction with nearby objects, whether one performs a goal-directed action, or wants to protect oneself from an incoming threat. Its physiological bases have been first discovered in non-human primates, where monkeys’ bimodal visuotactile neurons were described almost 40 years ago. These neurons, initially recorded from the ventral premotor cortex (area F4), respond to both tactile and visual stimuli within the immediate proximity of the monkey’s arm (Rizzolatti, Scandolara, Matelli, & Gentilucci, 1981a, 1981b), thus providing the PPS with a clear set of neurophysiological properties (see, for review, Brozzoli, Demattè, Pavani, Frassinetti, & Farnè, 2006; Brozzoli, Makin, Cardinali, Holmes, & Farnè, 2012; di Pellegrino & Làdavas, 2015; Làdavas & Serino, 2008; Makin, Holmes, Brozzoli, & Farnè, 2012). Based on these properties, a number of studies have proposed that a similar representation of PPS exists in humans: For example, many studies on cross-modal visual–tactile extinction, which we quickly recall below, have reported that this pathological sign may vary as a function of the distance of visual stimuli from the patients’ hands and that this PPS distance becomes larger after tool-use (Farnè & Làdavas, 2000; Maravita, Spence, Clarke, Husain, & Driver, 2000).

### Tool-use “extends” the peripersonal space

Over the last decades, several studies have focused on how tool-use could modify the PPS, from non-human primates to healthy humans, through neurological patients, the latter being principally in the domain of hemispatial neglect and extinction following right brain-damage. The first, extremely influential, study providing evidence in monkeys for tool-use to be capable of modifying the PPS has been reported by Iriki and colleagues (Iriki et al., 1996). Monkeys, who had been previously trained to learn using a rake-shaped tool, were conditioned to use the rake to retrieve for a few minutes some pieces of food that were otherwise out of their arm reach. By comparing the activity of parietal bimodal cells (i.e., cells responding to both tactile and visual stimuli, provided the latter originated from the immediate proximity of the hand-centred tactile receptive field) before and after the use of the rake, the authors found that, after tool-use, their visual receptive fields were enlarged along the tool axis. Despite the original interpretation emphasizing the tool-use-induced change in body schema (BS), these neurons’ responses were modified so as to cover the animal’s newly enlarged reaching space, functionally extended by the tool, thus testifying for the remapping of the PPS.

A similar form of representational plasticity has been revealed in humans as well, at first in neurological patients, through cross-modal visual–tactile extinction paradigms. These studies on right-brain-damaged patients focused on the patients’ inability to detect tactile stimulations, administered to their contralesional left hand, when a visual stimulus was delivered concurrently to their ipsilesional right hand. Numerous studies have established that this visual–tactile form of extinction is stronger when the visual stimulus is displayed in the peri-hand space (di Pellegrino, Làdavas, & Farnè, 1997; see, for review, Brozzoli et al., 2006, 2012). Most interesting for the present purposes, several studies additionally reported that, after a short period of active tool-use, cross-modal extinction was more severe for further distances, testifying for the extension of PPS (Farnè, Bonifazi, & Làdavas, 2005; Farnè, Iriki, & Làdavas, 2005; Farnè & Làdavas, 2000; Farnè et al., 2007; Làdavas, 2002; Maravita, Husain, Clarke, & Driver, 2001; Maravita & Iriki, 2004). Similar findings have been established thanks to studies on spatial neglect, with patients who experience deficits to report, orient, or respond to stimuli originating from the contralesional space, resulting in a rightward bias when asked to bisect lines (Ackroyd, Riddoch, Humphreys, Nightingale, & Townsend, 2002; Berti & Frassinetti, 2000; Neppi-Mòdona et al., 2007; Pegna et al., 2001). Berti and Frassinetti (2000) reported the first evidence for tool-use-dependent remapping of space in a case study of a neglect patient with a dissociation between near and far space following a stroke in the right hemisphere. This patient exhibited neglect symptoms in the near space, resulting in the usual rightward bias when performing the line bisection task with a laser pen, but not in the far space. Interestingly, when performing the task in the far space with a long stick allowing them to reach the line, the classical rightward bias re-emerged. Using the stick as a tool to expand the patient’s arm literally made the far space become near, possibly by extending the PPS to incorporate the stick in it. The opposite remapping direction – that is, the near space becoming far – is also possible, especially when using a tool that prevents *sensory continuity* with the target and activates the far space, such as a laser-pen (Neppi-Mòdona et al., 2007). Neppi-Mòdona et al. (2007) observed that when using such a tool, some neglect patients would remap the near space as being far in line bisection tasks (e.g., the neglect bias in the near space became as severe as in the far space). Complementarily, in a patient with a severe neglect in the near space they observed a reduced, even suppressed neglect bias when using a laser-pen that seemed inducing a dominance of far space coding. Near space coding implied a continuity between the tool and the target, visual feedback from this tool in the near space, and tactile proprioceptive feedback from the same tool in the far space. Conversely, the absence of these feedbacks indicated a far space representation (Neppi-Mòdona et al., 2007).

Neglect-like symptoms in the near space (or PPS) have been successfully induced in healthy subjects following a transient perturbation of the right posterior parietal cortex (rPPC), a region known to be activated during line bisection tasks (Fink et al., 2000; Fink, Marshall, Weiss, & Zilles, 2001), through transcranial magnetic stimulation (TMS; Bjoertomt, Cowey, & Walsh, 2002; Fierro et al., 2000; Fierro, Brighina, Piazza, Oliveri, & Bisiach, 2001; Giglia et al., 2015) or transcranial direct current stimulation (tDCS; Giglia et al., 2011). Crucially, Giglia and coworkers (Giglia et al., 2015) have recently reported that tool-use transfers the same TMS-induced neglect-like bias from the near towards the far space in healthy subjects, just as in neglect patients, thus testifying once again that the use of a long tool can remap the far space as nearer. In addition, this study provided direct evidence suggesting that the rPPC is fundamental for PPS representation (see also Serino, Canzoneri, & Avenanti, 2011).

The tool-use “power” of affecting different forms of spatial cognition does not only rely on patients, or patient-like evidence. Actually, changes in perception have been reported also in non-perturbed neurological (i.e., neurotypical) conditions. These effects have been attributed either to the preparation or intention to use a tool (Witt, Proffitt, & Epstein, 2005), or to lower level aspects of multisensory perception (see below). Witt et al. (2005) asked healthy participants to perform a perceptual matching task: A target was projected in front of them either beyond or within their reach, and they had to touch it, either with their hand or with a baton. For out-of-reach targets, subjects had to reach as far as they could. Then, they had to visually match the distance separating them from the first target. Targets were perceived as being closer to the body than they actually were when reached by the baton. More interestingly, no such effect was observed when participants were passively holding the baton and were not asked to do anything with it: Reachability influenced distance perception, but only when the subjects intended to reach for the target.

Hence, active tool-use appears to be necessary for PPS remapping, a finding that has been replicated several times (Anelli, Candini, Cappelletti, Oliveri, & Frassinetti, 2015; Farnè, Bonifazi, et al., 2005; Iriki et al., 1996; Maravita, Clarke, Husain, & Driver, 2002; Maravita, Spence, Kennett, & Driver, 2002; Serino, Bassolino, Farnè, & Làdavas, 2007). Only passively holding the tool is not sufficient to trigger changes in PPS (Anelli et al., 2015; Farnè, Bonifazi, et al., 2005; Farnè, Iriki, et al., 2005; Farnè & Làdavas, 2000; A. Rossetti, Romano, Bolognini, & Maravita, 2015; Witt et al., 2005), but it appears that it is the functional instead of the mere physical length of a tool that can modulate the extent of PPS elongation (Farnè, Iriki, et al., 2005). Peripersonal space remapping also appeared to be specific to experience and to be transient: Farnè and Làdavas (2000) reported that while PPS extension was observed immediately after tool-use in extinction patients, such effects were ephemeral, disappearing after a few minutes of passive holding of the tool. Similar findings were reported by Serino and others (Serino et al., 2007) in healthy but blindfolded subjects using a blind cane for a few minutes to explore the environment: When they were tested the following day, the previously extended PPS shrank back to its original dimension. In contrast, blind people who used the cane in everyday life had their PPS already extended towards the cane tip while holding it. Interestingly, when they were passively holding a weight-matched cane handle (i.e., not providing any gain in reachability), their PPS was limited to the area around their hand, just as in the healthy subjects before the cane-use. Such a specificity of tool-use-dependent plasticity of PPS has been recently corroborated by Bourgeois and colleagues (Bourgeois, Farnè, & Coello, 2014) who also demonstrated that a tool had to give a functional benefit to the arm in order to shape the PPS extent. They asked healthy subjects to perform a reachability judgement task, before and after using a tool, which could increase the arm length by either 60 cm (70-cm-long tool, 10-cm handle) or 0 cm (short tool 10 cm long, 10-cm handle). Reachability judgments were selectively modified by tool-use, subjects considering farther locations to be reachable only after using the long tool, whereas no modifications were observed when the arm length remained unchanged.

Despite initial agreement on the fact that active tool-use was necessary to trigger PPS extension, more recent studies have challenged this view. Mere tool-use observation, while passively holding the same tool, seems actually sufficient to remap the PPS (Costantini, Ambrosini, Sinigaglia, & Gallese, 2011). Studies by Brozzoli and colleagues (Brozzoli, Cardinali, Pavani, & Farnè, 2010; Brozzoli, Pavani, Urquizar, Cardinali, & Farnè, 2009) have actually revealed that changes in PPS may be observed when the bare hand moves to grasp a target object. Such an action-dependent modification of the PPS, as measured by the classical crossmodal congruency effect paradigm (Spence, Pavani, & Driver, 2004), takes place upon action starts and is further modulated during action execution, meaning that its remapping is regulated online, even when no tool-use is involved (Brozzoli et al., 2010, 2009). In addition, Serino and colleagues (Serino, Canzoneri, Marzolla, di Pellegrino, & Magosso, 2015) have recently developed a computational model able to simulate PPS neurons and behaviour in an audio–tactile interaction that allows for testing tool-use-dependent plasticity. They recreated the conditions of PPS remapping via tool-use using a simple audio–tactile training: A temporally correlated stimulation providing touch on the hand and auditory (or visual) input at a distance in space turned out to be sufficient to mimic the sensory–motor consequences of tool-use, hence causing an extension of the PPS. It thus appears that, more than the tool itself, the multisensory inputs that are typically generated by active tool-use are crucial to extend the PPS. Either active or passive, the functional experience with the tool seems ultimately to play a prominent role, as suggested by the gradual extension of PPS induced by the use of variable tool lengths (Farnè, Iriki, et al., 2005) and the durable modifications of the PPS that one can observe in “proficient” tool-users even in passive conditions (Bassolino, Serino, Ubaldi, & Làdavas, 2010). Notably, in the latter study the remapping after using or holding a computer mouse was observed only for the hand usually operating the mouse, but not the opposite hand, again suggesting a relatively high level of specificity in tool-use-dependent plasticity of the PPS.

From a theoretical perspective, de Vignemont and Iannetti (2014) have recently argued for the existence of two functionally distinct representations of PPS, the first being dedicated to protection of the body toward threats (the so-called “flight zone”), and the second to interactions with surrounding objects that the body wants to act upon (reaching/working space). In this context, tool-use seems to refer preferably to the latter, even if typical protective responses, such as the skin conductance response (SCR), can be modified by tool-use. In a recent study, Rossetti et al. (A. Rossetti et al., 2015, Experiment 1) measured this physiological variable for an incoming threat when a medical needle approached the subject’s hand: The anticipatory pain response was obviously highest when the needle actually touched the hand, and stayed high around the hand (5 cm), but was clearly smaller when the incoming threat was located in the far space (40 cm). Remarkably, after an active period of tool-use to retrieve some distant objects, the contact of the hand-held tool by the same needle provoked an opposite pattern on SCR modulation: The anticipatory pain response seemed to decrease close to the hand, but tended to increase for farther distances (20 and 40 cm), in such a way that the body responded to farther incoming threatening stimuli. This study may provide some evidence against an alternative interpretation of the PPS modulation, which posits that tool-use induces a shift of spatial attention to the functional tip of the tool (Holmes, 2012; Holmes, Calvert, & Spence, 2004, 2007; Holmes, Sanabria, Calvert, & Spence, 2007; Holmes, Spence, Hansen, Mackay, & Calvert, 2008), rather than a real extent of PPS. Indeed, SCR changes were found along the whole tool (and not only at the tip), and pure attentional training (e.g., passively holding the tool while detecting a missing target in a series of 15) did not extend the PPS, while active tool-use did (see also Bonifazi, Farnè, Rinaldesi, & Làdavas, 2007).

Overall, the ever-growing interest into the plastic properties of PPS has contributed a large body of evidence supporting the notion that tool-use does indeed modify the way our brain perceive the space around us. Yet, it has recently become clear that the PPS can be dynamically remapped by merely moving a bare hand (Brozzoli et al., 2010, 2009), and active tool-use is possibly not necessary to PPS remapping, as long as the synchrony of its sensorimotor consequences is preserved (Serino et al., 2007). When considering recent studies on the effects of tool-use on the body representation for action (i.e., the body schema), it seems reasonable to doubt as to whether tool-use interrogates specifically and solely the PPS representation. In what follows, we focus on tool-use approaches used to observe the plasticity of body representations.

## 2. Tool-use shapes the body

Besides changing the space around us by extending our peripersonal space, tool-use has been shown to modify the representation of our own body. In this section, after a non-exhaustive overview of different body representations, we pursue two main objectives. The first one is to testify about the benefits of tool-use as a paradigm to assess plasticity of body representations. The second aim is to suggest some of the main points one should take into consideration when using such tool-use-based approach to tackle body representations.

### Bodily representations: Body structural description, body schema, body image

As pointed out by several authors, while the body schema seems universally accepted and well described, with a relatively unifying definition, the other body representations (BR) remain conceptually and functionally difficult to disentangle. We chose here to mainly follow the conceptual analysis and criteria offered by de Vignemont (2010). Two main models have been proposed to try explaining the subdivision of the BR; first, the dyadic model dichotomizes the body schema (BS), a sensorimotor representation grounded into action, and the body image (BI), an action-free BR (Gallagher, 2005; Y. Rossetti, Rode, & Boisson, 1995). Second, the triadic model proposed by Schwoebel and others (Schwoebel & Coslett, 2005; see also Sirigu, Grafman, Bressler, & Sunderland, 1991), rather split the BI into two distinct representations: the body semantics, which constitutes the conceptual and linguistic level of body parts, and the body structural description (BSD), which is a visuospatial map of the body parts and their topological relationships. With that in mind, we chose here to label body representations (BR) the cognitive umbrella under which we then focus on three of them – namely, the body structural description, the body image, and the body schema. Indeed, these three BR have in common the fact of containing some spatial layouts of the body, a sort of spatial metrics that can potentially be affected by tool-use, which is absent in the body semantics. Additionally, we focus on these functionally specific BR as they have been shown to dissociate and rely on partially segregated neuroanatomical bases (Schwoebel, Buxbaum, & Coslett, 2004; Schwoebel & Coslett, 2005; Schwoebel, Coslett, & Buxbaum, 2001). Thus, we refer to BSD as the subdivision of the BI in the triadic model, and to the BI as the more general entity referred to by dyadic models.

### The body structural description (BSD)

Owing to the fact that, to our best knowledge, no previous study has assessed the effects of tool-use on the BSD, we only briefly refer to it in this review. Indeed, the BSD gathers structural information about location of body parts in the body and with respect to one another, in a way that is considered as mainly visuospatial and conscious (see Table 1). A few studies have attempted setting the neural bases of BSD in the left hemisphere, especially in the left temporal lobe (Schwoebel & Coslett, 2005) and in the posterior intraparietal sulcus (IPS; Corradi-Dell’Acqua, Hesse, Rumiati, & Fink, 2008; Corradi-Dell’Acqua, Tomasino, & Fink, 2009; Felician et al., 2004). However, all these authors agree on the difficulty in disentangling the neural bases of the different body representations, possibly due to the limited number of studies. For example, both BSD and the body image (see below) seem to be impaired following left temporal brain damage (Schwoebel & Coslett, 2005). A deficit in the BSD will cause some trouble in identifying human body parts on oneself, or others: the so-called auto- (or hetero-) topagnosia (Buxbaum & Coslett, 2001; Felician, Ceccaldi, Didic, Thinus-Blanc, & Poncet, 2003; Felician et al., 2004; Sirigu et al., 1991). A major role in building and updating this topological representation of the body is played by visual, as compared to proprioceptive and sensorimotor, input (Tessari, Ottoboni, Baroni, Symes, & Nicoletti, 2012). Thus, its dominant visual and conscious nature might be part of the reasons why the BSD has so far been neglected in the field of tool-use, which has given preference to either unconscious multisensory (PPS) or proprioceptive (BS) processes. Nonetheless, in the light of more recent evidence showing tool-use effects to impact also mainly visual and putatively conscious representations (e.g., the BI, Miller, Longo, & Saygin, 2014; see below), we would expect that the BSD, under yet unknown experimental circumstances, may also be affected by tool-use.

**Table 1.** Criteria used to qualify bodily and spatial representations.

	Peripersonal space	Body schema	Body image	Body structural description
Sensory inputs	Multisensory (vision, audition, touch, vestibular)	Proprioception Kinesthesis Touch	Multisensory (vision, audition)	Vision Verbal Somatosensory
Format	Multisensorimotor	Somatosensorimotor	Visuospatial	Visuospatial
Functional properties	Defensive movements Appetitive actions	Metric body knowledge for action (body parts’ position and size)	Body percept, concept, and affect; Visual metrics of the body	Structural information about body parts location
Accessibility	Mainly unconscious	Mainly unconscious	Conscious	Conscious

Note: Here we subdivide the body and space representation according to different criteria – namely, sensory inputs, format, functional properties and accessibility.

### The body schema (BS)

Since the introduction of the “postural schemata” by Head and Holmes (Head & Holmes, 1911; see also Bonnier, 1905), based upon a series of neuropsychological studies, the so-far most commonly used term for the body representation for action is body schema (see Table 1). Despite decades of confusion between labels and concepts among BS, body image, and peripersonal space (see, for discussion, Berlucchi & Aglioti, 2010; Cardinali, Brozzoli, & Farnè, 2009; de Vignemont, 2010), the BS has come to a relatively consensual definition: a highly plastic representation of the body parts, in terms of posture, shape, and size, that can be used to execute or imagine executing movements accurately (Medina & Coslett, 2010). The BS allows for execution and constant monitoring of our actions and appears to be fed mainly by proprioceptive, but also tactile and kinaesthetic, information (Head & Holmes, 1911; Shenton, Schwoebel, & Coslett, 2004). In terms of its neural underpinnings, the few available studies suggest that the BS depends on the activity of the somatosensory cortices, the intraparietal sulcus (Corradi-Dell’Acqua et al., 2009; Ehrsson, Kito, Sadato, Passingham, & Naito, 2005), and the dorsolateral frontal cortices (Schwoebel & Coslett, 2005). Thanks to the BS, we are capable of locating our body and its parts in space, knowing both where our left hand is, for example, and how to get there with our right hand, as well as whether our legs are stretched, bent, or crossed under the table (i.e., without vision). This information is refreshed instantaneously at every single movement of our body. Importantly, we typically become aware of the information carried out in the BS, as well as of their updating, only when we make a conscious effort. Otherwise, most of the BS activity and outcome is largely considered to occur unconsciously. Thus, the BS is essentially sensorimotor in nature. As such, many studies have tried characterizing the BS with either somatosensory or motor tasks. As a consequence, several paradigms have been proposed to test the BS, such as tactile localization and tactile distance perception tasks (Anema et al., 2009; Canzoneri et al., 2013; de Vignemont, Ehrsson, & Haggard, 2005), motor imagery (Schwoebel & Coslett, 2005), or ballistic movements (Y. Rossetti et al., 1995; Y. Rossetti, Rode, & Boisson, 2001). As we detail below, most of them have also been used to assess the plastic properties of the BS.

### The body image (BI)

Most typically brought in opposition to the BS (Berlucchi & Aglioti, 2010; de Vignemont, 2010), the BI has been conceptualized as a conscious, lexical, and semantic representation of the body and its parts, with their names and associated functions (Coslett, Saffran, & Schwoebel, 2002; Sirigu et al., 1991). The term BI, coined by Schilder (1935), has become the most accepted label for this BR, for which, however, consensus is not wide, and the proposed testing protocols may differ largely. Studies revealed damaged BI following temporal lesions (Schwoebel & Coslett, 2005). While in its initial conception the superficial schemata included the localization of stimulation on the body surface (Anema et al., 2009; Paillard, Michel, & Stelmach, 1983; but see Medina & Coslett, 2010), neuropsychological evidence indicated that the BI contains semantic knowledge about body parts and their function (Buxbaum & Coslett, 2001; Sirigu et al., 1991). At odds with the BS, the BI appears to rely heavily on visual inputs (see Table 1). Possibly for this reason, the term BI is more and more generally employed to refer to the visually based representation of the body shape and size – in other words, the visually based metrics of the body. In addition, BI is nowadays classically referred to in the rubber hand illusion studies (e.g., Kammers, Kootker, Hogendoorn, & Dijkerman, 2010), or other types of hand distortions (Medina, Khurana, & Coslett, 2015; Tamè, Farnè, & Pavani, 2011, 2013; Treshi-Marie Perera, Newport, & McKenzie, 2015) and body related disorders, such as the body integrity identity disorder (see, for a comprehensive view, Urgesi, 2015). Furthermore, in the last decade the BI has become of great interest in relation to patients with eating disorders, such as bulimia and anorexia nervosa (see Cicmil & Eli, 2014; Gardner & Brown, 2014; Lang, Lopez, Stahl, Tchanturia, & Treasure, 2014), a growing interest testified by the existence of a specific journal (*Body Image, An International Journal of Research*). Thus, the term BI has been put in relation to many other concepts, like body ownership and appearance, making the nosological problem of this BR and its relationships with the other BRs even more complex.

With this brief, taxonomic overview in mind, we now turn to our focus and interrogate the plastic features of these BR through the perspective of tool-use.

### Body schema and body image modifications by tool-use

#### Body schema

Our group has contributed converging evidence for BS plasticity after tool-use (Cardinali et al., 2011; Cardinali, Frassinetti, et al., 2009; Cardinali et al., 2012). Indeed, Cardinali, Frassinetti, et al. (2009) reported for the first time that the BS, in this case the unconscious arm representation as probed by execution of reach-to-grasp movements, was modified after using a mechanical grabber for 10 minutes. We recorded neurotypical participants’ free-hand movement kinematics before and after using this tool. Tool reach-and-grasp actions modified the subsequent free-hand movement kinematic profile, as reflected by the observation that participants took longer to reach both velocity and deceleration peaks, resulting in a longer movement time. In addition, the amplitude of both velocity and deceleration peaks was smaller, as compared to that observed during the bare hand movements performed before tool-use. Such changes did not occur for the grasping component of the movement (e.g., finger opening), but were limited to the transport one, thus suggesting a modification of the representation of the forearm, but not the hand. Subjects literally acted as if they had a longer arm after tool-use. Indeed, when compared to people with short(er) arms, participants with long(er) arms naturally displayed bare hand reach-to-grasp movements that were characterized by longer latencies, as well as reduced amplitudes, of velocity and deceleration peaks, with overall longer movement times. The kinematic pattern of long-armed people is thus similar to that observed after tool-use, irrespective of the real arm length (see Cardinali, Frassinetti, et al., 2009, Supplementary Material). These findings were taken as evidence for tool incorporation into the arm representation and, hence, plasticity of the BS.

Similar effects of “represented arm lengthening” were obtained when subjects were required to quickly point with their left index fingertip at anatomical landmarks of their right arm (elbow, wrist, middle finger) where an unseen tactile stimulus was delivered. After tool-use, subjects pointed to the wrist and elbow as if they were farther apart, hence they clearly relied on an extended arm length representation. Interestingly, their hand length representation, as indexed by the distance between the wrist and the middle fingertip end-pointing locations, remained unchanged. These results match the kinematics pattern well, with a modification of the transport component of the movement, while the grasping one was left unchanged (Cardinali, Frassinetti, et al., 2009).

More recently, we investigated the hypothesis according to which the mere mental imagery of tool-use could be sufficient to modify one’s arm’s length representation. Indeed, Fitt’s law, according to which the movement duration of an action increases with its difficulty, is also preserved in case of tool-use imagery (Macuga & Papailiou, 2012). Given that both actual and mental imagery of tool-use are known to alter space perception (Davoli, Brockmole, & Witt, 2012; Witt & Proffitt, 2008), we reasoned that tool-use imagery should trigger, on subsequent hand movement kinematics, similar effects to those induced by actual tool-use (Baccarini et al., 2014). In this study, we asked healthy participants to perform free-hand reach-to-grasp movements, before and after merely imagining performing this movement either with their hand (Day 1) or with a mechanical grabber passively held in their hand (Day 2). We ensured that the motor imagery was accurately performed by introducing changes in the orientation of the thumb–finger opposition axis needed to grasp the target object (after Frak, Paulignan, & Jeannerod, 2001), thus making the task more difficult for some orientations. The results showed that movement time was similarly affected by this manipulation of task difficulty in both actual and imagined reach-to-grasp movements. Crucially, after tool-use imagery participants displayed reduced amplitude peaks for the transport component (velocity and deceleration peaks), while there was no kinematic change after imagining to use the hand to execute the same movements (Baccarini et al., 2014). These results are similar to those observed after actual tool-use, which indeed consisted in the reduction in the maximal amplitude and protracted latency of some transport parameters, as well as the global lengthening of movement time (Cardinali, Frassinetti, et al., 2009). Taken together, these findings converge to indicate that imagining reaching out an object with a tool may be sufficient to trigger its plastic incorporation in the BS.

Noteworthy, participants were familiarized with the tool in the preceding day, and could see it while holding it, so both vision and proprioception were possibly at stake in producing the effects following mental tool-use imagery (Baccarini et al., 2014). In progress work in our laboratory, we are currently seeking for empirical evidence that the plasticity of BS could occur without recurring to any visual inputs, given that proprioception should in principle be sufficient to trigger body representation changes (Martel et al., 2014, Cognitive Neuroscience Society, CNS, abstract). This line of study is in keeping with the old tenet that proprioception and more generally somatosensation should allow a privileged access to the BS (Cardinali, Brozzoli, et al., 2009). In a related study, Cardinali and coworkers (Cardinali et al., 2011) asked healthy blindfolded participants to point quickly and accurately with their left index fingertip, to a specific location on their right arm that had previously been touched by the experimenter (finger, wrist, or elbow). In another condition, subjects had to verbally report where (on their right arm) they had been touched, by reading aloud the number on a ruler kept parallel to their (unseen) right arm in front of them. The distance between the different landing and read-out points was then calculated to assess the represented length of two body segments: the hand (fingertip–wrist distance) and the forearm (wrist–elbow distance), both before and after using the same mechanical grabber to perform reach-to-grasp movements. Participants’ performance revealed a significant effect of tool-use on both tasks, but only for the representation of forearm length; the hand length remained unchanged. When participants were making either conscious verbal judgements about, or unconscious movements toward, the tactile stimulation, the represented distance between the elbow and the wrist was extended after tool-use (Cardinali et al., 2011).

The neural bases of tool-use-dependent plasticity, whether during actual, imagined, or observed movements, have started to be investigated through functional magnetic resonance imaging (fMRI), electroencephalography (EEG), and positron emission tomography (PET) studies (Lewis, 2006). Although still limited in numbers, those studies have revealed the involvement of the frontoparietal cortices (Gallivan, McLean, Valyear, & Culham, 2013; Jacobs, Danielmeier, & Frey, 2010), especially the superior parietal lobule (SPL; Di Russo et al., 2006), and the left intraparietal sulcus (IPS; (Costantini et al., 2011; Tomasino, Weiss, & Fink, 2012; Valyear, Cavina-Pratesi, Stiglick, & Culham, 2007), or the posterior parietal cortex (PPC), which is known to integrate visual and somatosensory information (Inoue et al., 2001). Studies on monkeys highlighted an increase of grey matter in this same circuit after tool-use (Quallo et al., 2009). It is also believed that some neurons in area 5 of the parietal lobe could be the core of the BS, as they code for arm position through visual and somatosensory information in the monkey brain (Graziano, Cooke, & Taylor, 2000).

In the same way as PPS remapping, BS plasticity following tool-use relies on the functional importance of the tool to perform the action (Sposito, Bolognini, Vallar, & Maravita, 2012). In their study, Sposito and colleagues (2012) questioned the modification of the metric representation of the body by tool-use. They designed a forearm bisection task, consisting in estimating the midpoint of one’s own forearm before and after diversified tool-use training. After using a 60-cm-long tool, subjects indicated a more distal midpoint, according to the hypothesis of an increased arm length representation due to tool-use. Interestingly, after using a 20-cm-long tool, which did not substantially alter the reaching space, the described effect failed to appear, thus highlighting the importance of the gain in reachability provided by a tool as an essential component to lead to a BS update. The role played by the conscious feeling of ownership towards the arm using the tool has been revealed as critical, in some particular cases, as that of brain-damaged patients who suffer from a pathological embodiment of another person’s arm at the place of their own hemiplegic arm. Garbarini and colleagues (Garbarini et al., 2015) asked four of these patients to estimate the midpoint of their contralesional forearm before and after 15 min of tool-use training (similar to Sposito et al., 2012). When the patients were convinced to use the tool with their own paralysed arm (though they were merely looking at the experimenter arm + tool usage) they misjudged their forearm midpoint more distally (i.e., towards the hand), just as neurotypical participants did with respect to their real own arm. This finding definitively points to the existence of intimate relationships between the BR and the sense of ownership, as the possibility that tool-use affects the BS seems clearly conditional to the (possibly implicit) self-attribution of the effector acting with a tool. These authors, considering tool-use in terms of tool-embodiment, opened the field to studies about prosthetic arms, and the way they are integrated into the body representations, for an optimal sensorimotor control (Romano, Caffa, Hernandez-Arieta, Brugger, & Maravita, 2015). Prostheses can indeed be considered as tools in the sense that they allow the user to perform movements and actions otherwise not possible. Prosthesis use, as tool-use, changes the shape and size of the user’s body in an attempt to restore it to its original state by replacing a missing limb. If the concept of a tool becoming a body part has been around since the beginning of the nineteenth century, the idea that a prosthesis can become a body part has been accepted as true and obvious even more easily. The main issue with the research about embodiment is the quite vague definition of embodiment. Murray (2008) defines embodiment as “the way in which people experience their own body” (p. 127). This definition, while correct, includes many different phenomena and processes. More recently, de Vignemont (2011) proposed a systematic definition of embodiment that postulates the existence of three layers: motor embodiment (an object is embodied if it moves as a body part and is perceived as under one’s control); spatial (an object is embodied if the space it is located in is processed as body space); and affective (an object is embodied if the individual shows the same affective reactions as for his/her own body). When all the three aspects are present, one can talk about full embodiment.

A seminal work by Fraser (1984) used a kinematic approach to assess whether a prosthesis can become a body part. The rationale behind the study was that if the prosthesis is embodied it should be moved as a body part. Indeed, she found that the participant had a similar kinematic profile when performing movements with her intact arm as well as with her prosthesis. A few years later, McDonnell and colleagues (McDonnell, Scott, Dickison, Theriault, & Wood, 1989) investigated prosthesis embodiment in a group of congenital and acquired amputees by using a pointing task. They found that, when asked to point to the (unseen) position in space of their stump, amputees consistently overestimated the length of their arm but only while wearing a prosthesis. These two studies support the idea that prostheses can be embodied into the body schema– that is, they show motoric embodiment. However, they do not per se justify the claim that prostheses are fully embodied.

Interviews have been used to test affective embodiment (Murray, 2004, 2008). These studies reported that amputees can experience and describe various degrees of embodiment, which seems to be related to the amount of use of the prosthesis. Such relation between use and embodiment does not seem to be linear or unidirectional. Indeed, it seems that embodiment arises after a considerable amount of use, necessary to reach a level of comfort with the prosthesis. On the other hand, embodiment is suggested to play a role in supporting amputees in continuing using the prostheses. Pazzaglia and colleagues (Pazzaglia, Galli, Scivoletto, & Molinari, 2013; see also Galli, Noel, Canzoneri, Blanke, & Serino, 2015) developed a new comprehensive questionnaire to investigate wheelchair embodiment in patients with spinal cord injury. The 11 questions addressed different aspects of patients’ personal experience with the wheelchair, and a principal component analysis revealed that the majority of patients experience the tool as being part of their body. Interestingly, they also perceive it as a functional substitute for their legs but not as an external tool. That is, the wheelchair *is* the legs rather than a tool supporting or extending them.

Finally, a growing number of studies used a modified rubber hand illusion protocol where the participant stump and the prosthesis where synchronously or asynchronously stimulated (D’Alonzo, Clemente, & Cipriani, 2015; Ehrsson et al., 2008; Giummarra, Georgiou-Karistianis, Nicholls, Gibson, & Bradshaw, 2010; Rosén et al., 2009; Schmalzl, Kalckert, Ragnö, & Ehrsson, 2014). They reported that amputees can experience a rubber hand or a prosthesis as their own hand as shown by the fact that they feel the tactile stimulation on the stump as coming from the rubber hand/prosthesis and show physiological responses, as assessed with skin conductance response measures, to a threat to the prosthesis. These data show spatial and affective embodiment of prosthesis in amputees. However, the RHI-like paradigm might not be representative of real-life experience with a prosthesis. In such a quite artificial situation, amputees are not asked to control or act with the prosthesis, as they would in real life, but to passively observe it being brushed. As such, they speak more of whether a rubber-hand-like illusion can be induced using a prosthetic hand than of prosthesis embodiment on amputees, providing weak insight on whether and how embodiment can support prosthesis use and reduce rejection.

In addition, tool-use-dependent plasticity of the BS seems to be limb-specific, as recently suggested by Jovanov and colleagues (Jovanov, Clifton, Mazalek, Nitsche, & Welsh, 2015). In this study, participants were asked to look at a screen with a picture of a woman holding a rake, with the arm outstretched perpendicular to her body. Then they had to respond as fast as possible to some targets appearing on the hand, the tip of the rake, or on the foot of the woman, with either their own hand or their own foot. This task was performed before and after using a rake to grasp and move a tennis ball. After using the rake, subjects were faster to respond to targets appearing on the rake image, while they kept the same reaction time for foot- and hand-related targets.

Regarding the limb specificity, and as tool-use studies classically used rake or mechanical grabbers, one crucial aspect has been neglected for a long time: the tool morphology, as studied recently by Miller and colleagues (Miller et al., 2014). These authors referred to an implicit body representation, and even if they prefer the term “body model” (Longo & Haggard, 2010), a supposedly distinct representation from both the BS and the BI, we discuss its findings in terms of their relevance to the BS. In their study, Miller et al. (2014) asked subjects to perform a tactile distance judgement task (TDJ): They stimulated them in two distinct points, at two different locations (hand/arm and forehead), and asked participants to judge where the distance was bigger. Although clearly conscious and perceptive, the fact that the input was tactile and allowed for accessing to the metrics of the body led us to consider this task as assessing, at least partly, the BS. The TDJ was performed before and after using a tool to grasp a balloon: either a classical mechanical grabber or a hand-shaped tool. Crucially, after using the classical arm-shaped grabber, tactile distance perception on the hand was not modified, while that on the arm was. Conversely, after using the hand-shaped grabber, only tactile perception on the hand was modified. Those changes were opposite in direction: increased width and decreased length of the hand/the arm. These findings highlight that tool morphology itself, linked with the functional role of the tool, is crucial in the updating of the BS.

Several studies (Baccarini et al., 2014; Cardinali, Frassinetti, et al., 2009; Martel et al., 2014, CNS abstract) employing tools whose basic property was to lengthen the participants’ arm, have found kinematic changes following tool-use that were limited to the reaching component of the movement. This consistently replicated finding reinforce the idea that with an arm-lengthening tool, the representation of the arm is selectively modified. The changes in the hand perceptual representation reported by Miller et al. (2014) after using a hand-widening tool could thus be concomitant with kinematic changes that could be selective of the grasping part of the movement. Results from our group confirm this prediction: When the distal length of the thumb and index fingers is lengthened, subsequent free-hand prehensile movements are altered selectively with respect to the grasping component of the movement, leaving the transport component unchanged (Cardinali, Brozzoli, Finos, Roy, & Farnè, 2016). One finding that deserves discussion is that the reduced length in the arm perceptual representation reported by Miller and colleagues after the use of the long grabber does not readily fit the increased arm length representation described by the post-tool kinematics (Baccarini et al., 2014; Cardinali, Frassinetti, et al., 2009; Martel et al., 2014, CNS abstract) or the post-tool somatosensory driven pointing (Cardinali et al., 2011; Cardinali, Frassinetti, et al., 2009). Miller and collaborators have suggested that this discrepancy could be the result of the difference between the tasks across studies. Critically, to our best knowledge, the only other study that observed a shortening of perceived arm length after tool-use (Ganesh, Yoshioka, Osu, & Ikegami, 2014) required, once again, the conscious participants’ estimation of the size of their effectors. They observed that what they suggest to be the earliest stage of tool incorporation goes with a shortening of the perceived arm length, which then turns into the previously reported lengthening of perceived arm length with protracted tool-use.

The task used by Miller (Miller et al., 2014) was originally used by de Vignemont and colleagues (de Vignemont et al., 2005) who determined that a vibration of the biceps led to the subjectively experienced extension to the right arm. Recently Tajadura-Jiménez and colleagues (Tajadura-Jiménez et al., 2012) showed evidence of BS updating by introducing an audio-tactile conflict. Participants had to tap on the floor with their fist at different locations, and the tapping sound was manipulated to originate from different distances. If this task recalls the audio-tactile synchrony paradigms used by Serino and his colleagues (Serino et al., 2015), hence possibly involving an extension of the PPS, Tajadura-Jiménez and collaborators used a tactile distance judgement task akin to that used by Miller and colleagues (Miller et al., 2014) to assess the BR. When the sound came from twice the distance from the tapping action, the tactile distance task was biased in the direction of an elongated BR. These findings were recently extended to investigate the role played by agency and kinaesthesia in producing these effects (Tajadura-Jiménez, Tsakiris, Marquardt, & Bianchi-Berthouze, 2015). Overall, one may wonder whether a conscious estimation task could also influence the BS after tool-use, as it seems to influence the PPS and possibly other forms of body elongation after synchronous somatosensory and auditory stimulation at a distance from the body. The study reported in the PPS section above by Serino and colleagues (Serino et al., 2015) showed that PPS extension (similar to what typically induced by tool-use) could also be obtained by mere audio-tactile synchrony. It thus seems hard to clearly qualify which BR is selectively tackled by these multisensory-stimulation-based approaches. Other studies have highlighted that tool-use can indeed shape both PPS and BS when both were tested at a perceptual level (Canzoneri et al., 2013), although the similar outcome does not necessarily call for an identity between these representations and cognitive processes (see, for a dissociation, Bassolino, Finisguerra, Canzoneri, Serino, & Pozzo, 2014). One further possibility is that once directly modified by tool-use, the change induced within the BS can spill over the BI, or/and the PPS (Cardinali, Brozzoli, et al., 2009; Cardinali et al., 2011).

#### Body image

In our view, the rubber hand illusion (RHI) is to the body image what tool-use is to the peripersonal space. Until recently, the BI has mainly been referred to the concept of body ownership in studies of the RHI, which consists in tricking the subjects to believe that a fake rubber hand is their own, by brushing their unseen hand synchronously with a fake hand (see, for review Kilteni, Maselli, Kording, & Slater, 2015; Tsakiris, 2010). Again, BI and PPS have become a common arena for framing influential theoretical models of how the body is owned and represented in the brain (Blanke, Slater, & Serino, 2015; Makin, Holmes, & Ehrsson, 2008). However, as tool-use started to “dig its way” as a paradigm to interrogate other BR such as the body schema, the interest started to grow regarding the relationships between tools, their use, and the BI, particularly since it seemed difficult to feel ownership over non-hand-shaped tools (Tsakiris, Carpenter, James, & Fotopoulou, 2010; de Vignemont & Farnè, 2010), a limit that could possibly extend to enacted, but not necessarily self-sensed, robotic hands (Romano et al., 2015). In this rich but complex theoretical framework, the BI initially appeared to be immune to tool-use. For example, in the work by Cardinali and colleagues alluded to above (Cardinali et al., 2011), there were two additional conditions that, by manipulating the type of the input and output task modalities, were designed to tackle more specifically the BI. In one condition, blindfolded subjects had to point quickly and accurately with their left index finger to a specific location on their right arm that, instead of being signalled by tactile stimulation, was named by the experimenter (“finger”, “wrist”, or “elbow”). In another condition, subjects had to verbally report the position of the named anatomical landmark by reading aloud the number corresponding to these landmarks from a ruler that was visible in front of them. The distance between the landing and read aloud locations was then calculated to assess the represented length of arm and hand, both before and after using the same long mechanical grabber. Results showed no change in the arm (or hand) representation after tool-use in either condition. Noteworthy, the same subjects actually showed significant changes of arm (but not hand) length estimation in the two conditions where touch was used as input modality to indicate the anatomical landmarks, and thus designed to tackle the BS (Cardinali et al., 2011). Overall, this pattern of results suggests that tool-use had no consequences on the conscious representation of the body (e.g., the BI).

Among the main features of the BI, besides that of being typically accessed via conscious explicit tasks, there is its heavy dependence upon vision. Miller and colleagues (Miller, Longo, & Saygin, 2015, Vision Sciences Society, VSS abstract) addressed this issue by asking participants to judge whether a depicted hand was wider than their own, or not, before and after practising with a hand-shaped tool that was much bigger than average human hands. When tool-use was performed with visual feedback, most of the subjects judged that they had a wider and shorter hand when retested after use of such a tool. In contrast, when blindfolded participants used the mechanical hand, no modification of the conscious hand representation occurred afterwards. Hence visual feedbacks could be necessary to update the BI. However, we anticipate that this debate is far from being over, as, for example, the RHI has been observed even without visual input, when blindfolded subjects reported the impression of stroking their own hand while the experimenter was stroking it and at the same time using their other hand to touch a rubber hand (Ehrsson, Holmes, & Passingham, 2005).

When turning to consider the neural underpinnings of the BI, at least under the umbrella of the concept of self-awareness, many studies have pointed at the right temporo-parietal junction as one of the best candidate regions. Lesions in this area may result in neglect of the left side of the body (Mort et al., 2003) and anosognosia for hemiplegia (Berlucchi & Aglioti, 1997). More recent studies have the pointed to other regions that may be critical for BI as somatoparaphrenia, the delusional beliefs towards one’s own affected limb, have been observed with lesions in deep cortical and subcortical grey structures (thalamus, basal ganglia, amygdala), as well as in medio-frontal regions, such as the middle and inferior frontal gyrus, postcentral gyrus, or temporo-parietal areas (Feinberg, Venneri, Simone, Fan, & Northoff, 2010; Gandola et al., 2012; Invernizzi et al., 2013; Romano, Gandola, Bottini, & Maravita, 2014; Vallar & Ronchi, 2009). Right posterior insula also appears involved when the sense of limb ownership is evoked (Baier & Karnath, 2008). Direct electrical stimulation of the right temporo-parietal junction (rTPJ) in a neurosurgical patient elicited experiences of seeing her body from an external perspective (“out-of-body experience”), and of illusory transformations of the arms and legs (Blanke, Ortigue, Landis, & Seeck, 2002). Based on these results, rTPJ may be the neural source of a model of one’s own body, against which multisensory stimuli are tested. To test this hypothesis, Tsakiris, Costantini, and Haggard (2008), delivered transcranial magnetic stimulation (TMS) immediately after synchronous visuotactile stimulation to investigate the role of this area in the processing of sensory events during RHI (Tsakiris et al., 2008). Overall, TMS over rTPJ reduced the extent to which the rubber hand was incorporated into the mental representation of one’s own body, and it also increased the incorporation of a neutral object (e.g., a spoon), as measured by the proprioceptive drift towards or away from the viewed object. An object (i.e., a rubber hand) that would normally have been perceived as part of the subject’s own body was no longer significantly distinguished from a clearly neutral object, suggesting that the disruption of neural activity over rTPJ blocked the contribution of the body-model in the assimilation of current sensory input, making the discrimination between what may or may not be part of one’s body ambiguous. While an exhaustive review of this line of research is outside the scope of the present review, we refer the interested reader to a recent authoritative review on the subject of bodily self-consciousness (Blanke et al., 2015).

## General discussion

As mentioned in the introduction, this review was aimed at addressing two main issues: testifying of the benefits of tool-use as a paradigm to assess body representation plasticity, and to highlight the major points we should take into consideration when studying body representations.

### Tool-use, a tool to assess body representations

We have tried here to gather the available pieces of evidence suggesting that tool-use allows for the access to body representations and their plasticity. Several studies converge in indicating that, when used in real life, or even merely by mental imagery, tools can be incorporated in what we have identified as the body schema (Baccarini et al., 2014; Cardinali et al., 2011; Cardinali, Frassinetti, et al., 2009; Cardinali et al., 2012). In this respect, it is important to notice that in all these studies, the object to be grasped with either the tool or the hand was always located inside the reaching space of the arm. Far from being a methodological detail, the fact of acting in the same space with the tool and the hand allows us to isolate tool-use specific effects, ruling out possible confounds due to the fact of acting with either effector in different sectors for space. Tool-use remapping also appears to be limb-specific (Cardinali et al., 2016; Jovanov et al., 2015) and dependent upon the function and morphology of the tool (Cardinali et al., 2016; Miller et al., 2014) to perform a specific action (Sposito et al., 2012).

Some interesting questions remain to be addressed. A major question is related to the dynamics of the BS updating. Ganesh and colleagues (Ganesh et al., 2014) testified of a fast initial reduction of the arm representation, which is then followed by the repeatedly reported extension of BS. Whether this apparent discrepancy is entirely attributable to the differences in the tasks used remains unclear, and future investigations are awaited to elucidate the temporal and spatial dynamics of the processes contributing to tool embodiment (see, for review, de Vignemont, in press). Last, but not least, all the effects that have been reported so far following tool-use are (supposedly) temporary. This assumed transient character can actually be contrasted by what seems to be a rather permanent change in blind cane expert users (Serino et al., 2007), and prolonged tool-use may actually translate into more permanent neural changes of BRs. Yet, if we readily accept the term “plasticity” to deal with such tool-use-dependent effects, we should probably think in terms of plasticity also when those effects disappear. This spatio-temporal plasticity remains so far unexplored: Is the shrinking back gradual, or immediate like an elastic band going back to its default size (de Vignemont & Farnè, 2010), as has been observed for the peripersonal space retraction in adults and children (Caçola & Gabbard, 2012)?

### Tool-use, a useful yet not universal tool

Other important questions remain unanswered, especially on how to test the BI to know for sure whether it is immune to tool-use or not. Cardinali and colleagues (Cardinali et al., 2011) tried identifying which combination of inputs and outputs was needed to exclusively or preferentially assess body representations: A pure motor task will allow access to the BS, and be sensitive to tool-use, while a pure perceptual and verbal task will trigger the BI, with no mark of tool-use. Strikingly, with a verbal input and a motor output, no effect of tool-use was observed, suggesting that this combination provides marginal or no access to BS, being possibly more related to BI. Conversely, a tactile input with a perceptual output was sufficient to make the key signature of tool incorporation visible. This finding may suggest that such a combination is sufficient to reveal that BI is also sensitive to tool-use. An alternative interpretation is, however, possible – namely that the BS, once affected by tool-use, may have mediated this plastic change into the BI. This consideration also raises the point of the possible functional interactions between BRs, which remain, however, outside the scope of the present review (see Kammers et al., 2010).

Despite some overlap in the way they can be updated (Canzoneri et al., 2013) and, to some extent, in the brain areas involved, the PPS and the BS are possibly distinguishable through their sensory inputs and functional properties (Cardinali, Brozzoli, et al., 2009). Bassolino and others (Bassolino et al., 2014) recently gave one of the first insights into the dissociable plasticity between PPS and BR. In a paradigm of arm immobilization, they investigated how differently or similarly the PPS and the BR would react to non-use or over-use of the arm. Through several tasks, they found that PPS representation did not change for the over-used arm and was reduced for the non-used arm, whereas BR of the arm was modified after overuse but could not shrink after non-use. This reinforces the hypothesis that, as the body cannot biologically shrink, the BS can only extend, but never changes in the opposite direction (Bassolino et al., 2014; Cardinali, Brozzoli, et al., 2009; de Vignemont et al., 2005; but see Ganesh et al., 2014; Miller et al., 2014). Several studies have established that after surgical extension of limbs in achondroplasic patients, body representations, namely the BS and the explicit BI, were modified toward a “normalization” when compared to body-typical subjects (Cimmino et al., 2013; Di Russo et al., 2006). The PPS also appeared enlarged (Cimmino et al., 2013), as the physical length of the arm is known to correlate with some spatial processes at a distance (Longo & Lourenco, 2007). Thus, we think we should be careful when choosing the tasks, inputs, and outputs that we use when studying body and space representation plasticity.

Before concluding, we wish to list some of what we think are amongst the most urgent questions and issues that future research efforts should consider. A first obvious question that remains open is the extent to which a single tool-use paradigm (though already enriched by many variants so far) can probe each and all of the different BRs. While several findings converge in showing that tool-use can contribute to the study of the BS with some degree of specificity, new studies are needed to provide more definitive answers with respect to the BI. For certain, the so-far neglected susceptibility of the BSD to the tool-use paradigm should be explored. The principled motivation for a systematic approach of tool-use as a model paradigm for the study of body representations resides, in our view, in the fact that using the same paradigm to approach different BR will, first, elucidate whether they are generally or rather specifically tackled by this manipulation; second, and most important, such a systematic approach would have the merit of contributing to define the critical conditions to be met for a given BR to become, if possible, sensitive to the representational plasticity induced by tool-use. In that, tool-use could keep the promise of opening a window into the study of body representations and their plasticity. Last, but not least, the systematic questioning of BR via tool-use paradigms can help refining the current models that we use to frame these BR, by clarifying in more mechanistic terms their operational definitions.

To conclude, we believe that tool-use, despite the growing interest in the last years, still has many things to offer, especially in the study of body representations and their plasticity. Its specificity and selectivity to affect different BR may empower our capability to disentangle the still unsolved questions about body representations and their plastic modification.
